# Pneumatosis cystoides intestinalis in dermatomyositis: a case series report and literature review

**DOI:** 10.3389/fimmu.2023.1194721

**Published:** 2023-05-23

**Authors:** Jianwen Liu, Ling Zhang, Shuo Chen, Xin Lu, Shanshan Li

**Affiliations:** ^1^ Department of Rheumatology and Immunology, Fujian Provincial Hospital, Shengli Clinical Medical College of Fujian Medical University, Fuzhou, China; ^2^ Department of Radiology, China-Japan Friendship Hospital, Beijing, China; ^3^ Department of Gastroenterology, China-Japan Friendship Hospital, Beijing, China; ^4^ Department of Rheumatology, Key Laboratory of Myositis, China-Japan Friendship Hospital, Beijing, China

**Keywords:** pneumatosis cystoides intestinalis, dermatomyositis, asymptomatic, pathological mechanism, prognosis

## Abstract

Pneumatosis cystoides intestinalis (PCI) in adult dermatomyositis (DM) is rarely described. This report aimed to describe the clinical features and prognosis of PCI in six adult patients with DM (four with anti-MDA5 antibodies, one with anti-SAE antibodies, and one with anti-TIF-1γ antibodies). Except for one patient with transient abdominal pain, the remaining five patients were asymptomatic. PCI occurred in the ascending colon in all patients, of whom five had free gas in the abdominal cavity. No patients received excessive treatment, and PCI disappeared in four patients during the follow-up. Additionally, we reviewed previous studies on this complication.

## Introduction

Dermatomyositis (DM) is a chronic autoimmune disease characterized by skin or muscle damage and may involve the lungs, gastrointestinal (GI) tract, and other organs (1). Previous reports showed that 22%–50% of patients with DM have GI involvement (2–4). The most common GI manifestation of DM is dysphagia, occasionally accompanied by an ulcer, hemorrhage, or perforation (5, 6). Furthermore, pneumatosis cystoides intestinalis (PCI) is a rare manifestation of GI involvement, which is less reported in adult patients with DM (7). This complication is characterized by gas accumulation in the intestinal wall. PCI can occur anywhere within the GI tract, and most patients with PCI are asymptomatic (8, 9). However, patients with PCI may be misdiagnosed as having an acute abdominal disease due to the presence of free gas in the abdominal cavity when the vesicles rupture in certain conditions. Therefore, they will undergo some excessive examination and treatment.

This report aimed to clarify the characteristics of this rare manifestation in adult patients with DM and report the clinical features and prognosis of PCI in this case series. All patients in this case series fulfilled the 2018 European Neuromuscular Centre classification criteria (10). Furthermore, previous literature on this complication was analyzed, which could broaden our understanding.

## Case series report

### Case 1

A 60-year-old woman was diagnosed with DM in June 2019. She had heliotrope sign, Gottron’s papule, severe muscle weakness, dysphagia, and anti-SAE antibodies. She received pulse therapy of methylprednisolone (MP) 240 mg daily for 3 days and then MP 40 mg daily, combined with methotrexate (MTX) 10 mg per week as initial treatment.

**Figure 1:**
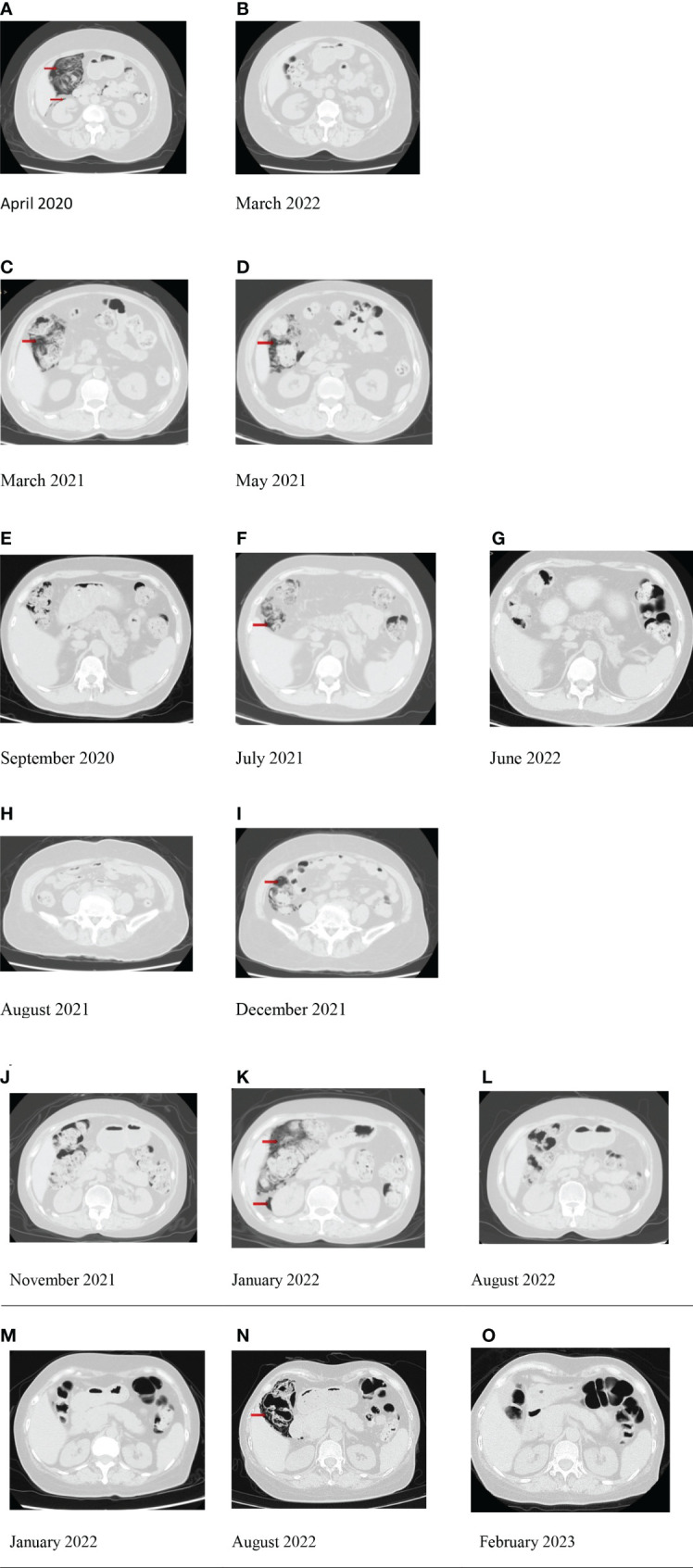
The computed tomography (CT) images of pneumatosis cystoides intestinalis (PCI) in six dermatomyositis (DM) patients. Red arrow: pneumatosis cystoides intestinalis and free gas in the abdominal cavity. **(A)** PCI in case 1; **(B)** the disappearance of PCI in case 1. **(C)** PCI in case 2; **(D)** the reduction of PCI in case 2. **(E)** The prior CT without PCI in case 3; **(F)** PCI in case 3; **(G)** the disappearance of PCI in case 3. **(H)** The prior CT without PCI in case 4; **(I)** PCI in case 4. **(J)** The prior CT without PCI in case 5; **(K)** PCI in case 5; **(L)** the disappearance of PCI in case 5. **(M)** The prior CT without PCI in case 6; **(N)** PCI in case 6; **(O)** the disappearance of PCI in case 6.

In April 2020, she was admitted to our department due to the aggravation of muscle weakness (MMT8 score 50) and rash. The C-reactive protein (CRP) level was 0.284 mg/dl (normal range: <0.8 mg/dl). Chest computed tomography (CT) scan showed the presence of interstitial lung disease (ILD), manifested as organizing pneumonia (OP) and non-specific interstitial pneumonia (NSIP). Muscle magnetic resonance imaging (MRI) showed mild inflammatory signals in lower limb muscles. She underwent an abdominal CT scan for tumor screening, which showed thickening of the intestinal wall, free gas in the abdominal cavity, and gas in the colon wall (**Figure 1A**). She had no abdominal symptoms. PCI was considered. MTX was switched to tofacitinib 5 mg twice daily in the treatment due to the activity of the disease. She continued to use prednisone (Pred) 35 mg daily. After the treatment, her rash and muscle strength improved. In March 2022, abdominal CT re-examination showed the disappearance of PCI (**Figure 1B**).

### Case 2

A 61-year-old man was diagnosed with DM in August 2020. He had heliotrope sign, Gottron’s papule, severe muscle weakness, dysphagia, and anti-TIF-1γ antibodies. Initially, he received pulse therapy of MP 500 mg daily for 3 days and MP 40 mg daily combined with MTX 10 mg per week. His symptoms improved.

In March 2021, the patient was admitted to our hospital for the first time due to worsening muscle weakness (MMT8 score 46). He had a normal CRP level (<0.1 mg/dl). Chest CT scan showed the presence of mild ILD, manifested as OP. His muscle MRI showed inflammatory signals in the lower limb muscles. He underwent an abdominal CT scan for tumor screening, which showed a significant accumulation of free gas in the abdominal cavity and gas in the colon wall (**Figure 1C**). He had no abdominal symptoms or signs of peritonitis. PCI was considered, and no excessive treatment was given. Considering the relapse of DM, a combined treatment of MP 40 mg daily, baricitinib 4 mg daily, and intravenous immunoglobulin (IVIG) was administered. In May 2021, abdominal CT re-examination showed the disappearance of free gas and the decrease of gas in the colon wall (**Figure 1D**).

### Case 3

A 49-year-old man was diagnosed with DM in September 2020 (**Figure 1E**). He had fever, Gottron’s papule, ILD, and anti-MDA5 antibodies. He was initially treated with MP 60 mg daily and tacrolimus (TAC) 1 mg twice daily.

In July 2021, the patient was admitted to our department for regular visits. At the time, he did not have any rash. Laboratory tests, including routine blood tests, muscle enzyme levels, and inflammatory markers, were within the normal range. The serum ferritin level was 166.5 ng/ml (normal range: 23.9–336.2 ng/ml). Additionally, the titer of the anti-MDA5 antibodies was also negative. He underwent a chest CT scan as a routine examination, which showed a stable condition of NSIP and a small amount of free gas in the abdominal cavity by accident. Abdominal CT scan showed gas accumulation in the colon (**Figure 1F**). PCI was diagnosed, and no excessive treatment was given for PCI. He continued to take MP 12 mg daily and TAC. In June 2022, abdominal CT re-examination showed the disappearance of PCI (**Figure 1G**).

### Case 4

A 69-year-old woman was diagnosed with DM in our department in August 2021 (**Figure 1H**). She had heliotrope sign, Gottron’s papule, perinail, erythema, mild muscle weakness, ILD, and anti-MDA5 antibodies. She was initially treated with MP 40 mg daily and tofacitinib 5 mg twice daily, and her clinical symptoms improved.

In December 2021, she suffered from a skin infection and was admitted to our department. She received MP 16 mg daily and tofacitinib. She had mildly higher levels of serum ferritin (499.7 ng/ml) and lactate dehydrogenase (LDH) (332 U/L) (normal range: 100–250 IU/L). Other laboratory tests, including the cytokines and routine blood tests, were within the normal range. She underwent a chest CT scan which showed a stable condition of NSIP, a small amount of free gas in the abdominal cavity, and gas accumulation in the ascending colon (**Figure 1I**). The patient complained of temporary abdominal pain for several minutes before hospitalization, and her symptoms improved. Physical examination showed no signs. After consulting with a gastrointestinal surgeon, a rupture of PCI was diagnosed. Antibiotics were given due to the skin infection, and tofacitinib was switched to cyclosporine A (CsA) 75 mg twice daily.

### Case 5

A 59-year-old woman was diagnosed with DM in our department in November 2021 (**Figure 1J**). She had polyarthritis, Gottron’s papule, rapid progressive ILD, and anti-MDA5 antibodies. She was initially treated with MP 80 mg daily and CsA 50 mg twice daily, and her clinical symptoms improved.

In January 2022, the patient was admitted to our department for a routine visit. She had a normal range of routine blood tests and inflammatory markers. The LDH level was slightly higher (349 U/L), and the concentration of CsA was within the normal range. She underwent a chest CT scan, which showed a stable condition of NSIP and large gas accumulation in the ascending colon. Abdominal CT was performed for confirmation and evaluation immediately. It showed a small amount of free gas in the abdominal cavity and gas in the colon wall (**Figure 1K**). Intestinal perforation was initially considered. Fasting and taking antibiotics were prescribed for 1 day at first. However, the patient had no symptoms or signs of peritonitis. The final diagnosis was PCI based on clinical signs and images. No treatment was performed for PCI. She continued to take MP 12 mg daily and CsA. In August 2022, abdominal CT re-examination showed PCI remission (**Figure 1L**).

### Case 6

A 57-year-old woman was diagnosed with DM in January 2022 (**Figure 1M**). She had heliotrope sign, Gottron’s papule, perinail, erythema, mild muscle weakness, ILD, and anti-MDA5 antibodies. She was initially treated with MP 40 mg daily and TAC 1 mg twice a day as the initial treatment. She complained of constipation during the hospitalization period, and abdominal CT and enteroscopy showed no abnormality.

In August 2022, the patient was hospitalized again to assess the disease activity. Laboratory tests, including routine blood tests, muscle enzyme levels, and serum ferritin levels, were in normal ranges. Additionally, the titer of anti-MDA5 antibodies was negative. She underwent a chest CT scan, which showed a stable condition of OP and NSIP and a large gas accumulation in the ascending colon wall by accident. Abdominal CT scan also showed gas in the colon wall (**Figure 1N**). The patient was asymptomatic, and no signs of peritonitis were observed. The patient was finally diagnosed with PCI. As the disease was stable, she continued to take MP 6 mg daily and TAC. In February 2023, abdominal CT re-examination showed PCI remission (**Figure 1O**).

Although the clinical characteristics of the six patients were different, they also had some common characteristics. They all had diabetes. Additionally, PCI in all patients was located in the ascending colon. A summary of some other clinical features is shown in **Table 1**.

**Table 1:** The clinical features of 18 patients with PCI.

Case	Age (years), sex	Disease duration (months)	Disease activity	Past history	GI tract presentation before the onset of PCI	Symptoms at PCI onset	PCI region	Free air in the abdominal cavity	PCI treatment	Prognosis	Pathological
P1/1993 (11)	ND, F	ND	Stable	ILD	ND	ND	ND	Yes	ND	ND	None^a^
P2/1999 (12)	61, F	17	Stable	No	Hiatal hernia + atrophic gastritis	Abdominal pain + abdominal distension	Ileum	Yes	Surgery	Remission	None^b^
P3/2004 (13) (Mi-2)	58, F	8	Active	No	Dysphagia	No	Ascending colon	Yes	Fasting	Remission	ND
P4/2005 (14)	69, F	120	Stable	No	No	Abdominal distension	Jejunum, ileum	Yes	Oxygen therapy	Remission	None^c,d^
P5/2006 (15)	53, F	48	Active	Diabetes (voglibose)	Dysphagia	Abdominal distension	Ascending colon, descending colon	Yes	Termination of AGI	Remission	ND
P6/2008 (16)	53, F	3	Active	No	Constipation	Abdominal pain	Jejunum	Yes	Oxygen therapy, bowel rest, antibiotics	Remission	ND
P7/2008 (17)	30, F	84	Stable	No	Dysphagia + constipation	Abdominal distension	Small intestine, ascending colon	Yes	Fasting	Remission	ND
p8/2012 (18)	67, F	12	Stable	No	No	Diarrhea	Colon	Yes	Oxygen therapy, fasting, antibiotics	Remission	ND
P9/2012 (7)	59, F	36	Stable	ILD	No	Abdominal distension	Colon	Yes	Antibiotics	Remission	ND
P10/2012 (7)	41, F	240	Active	ILD	No	Abdominal pain	Ileum	Yes	Surgery	Death/ARDS	None^e^
P11/2013 (19)	51, F	32	Active	No	No	Nausea + vomiting + fever	Transverse colon	Yes	Surgery, oxygen therapy, fasting, antibiotics	Remission	None^f^
P12/2017 (20)	70, M	ND	Active	No	Dysphagia	Abdominal distension	Ascending colon	Yes	Oxygen therapy, fasting, antibiotics	Death/infections	None^g,h^
P13 SAE	60, F	10	Relapse	Diabetes (acarbose)	Dysphagia	No	Ascending colon	Yes	No	Remission	ND
P14 TIF1r	61, M	7	Relapse	Mediastinal emphysema Diabetes (acarbose, sitagliptin)	Dysphagia	No	Ascending colon	Yes	No	–	ND
P15 MDA5	49, M	10	Stable	Diabetes (acarbose)	No	No	Ascending colon	Yes	No	Remission	ND
P16 MDA5	69, F	4	Stable	Diabetes (insulin) Skin infection	No	Temporary abdominal pain	Ascending colon	Yes	No	–	ND
P17 MDA5	59, F	2	Stable	Mediastinal emphysema Diabetes (acarbose, sitagliptin)	No	No	Ascending colon	Yes	Fasting and antibiotics for 1 day	Remission	ND
P18 MDA5	57, F	7	Stable	Diabetes (acarbose, metformin, linagliptin) Endoscopy	Constipation	No	Ascending colon	No	No	Remission	ND

AGI, alpha-glucosidase inhibitor; None, no vasculitis; ND, no data.

a Ischemic changes were seen but biopsy specimens did not show vasculitis.

b The resected bowel showed typical histological features of pneumatosis cystoides intestinalis with a giant cell reaction around the gas-filled cysts. No evidence of vasculitis or intravascular thrombosis.

c Colonoscopy was performed to examine the colon and ileum. Although the colon and rectum were intact, polypoid lesions like submucosal tumors (SMTs) with redness of the mucosa were seen in the ileum (approximately 30 cm beyond the ileum end). The polyps were elastic and movable, and biopsy specimens showed non-specific inflammation with no air-filled cysts.

d Endoscopic pathological.

e Both macro- and microscopic findings revealed that the muscularis propria was disrupted and replaced with fat tissue. There was no pathological finding of abscess or vasculitis.

f Colon histopathology. Cystic spaces within the submucosa and subserosa are focally lined by histiocytes, a classic feature of pneumatosis intestinalis. Submucosal cystic dilation typical of pneumatosis intestinalis is present. Lymphoid aggregate is normal in appearance. PCI was found unrelated to infection, malignancy, or vasculitis, and no frank perforation was present.

g No abnormalities, such as gas or fistula, were evident in the small or large intestines on histological examination at autopsy.

h Autopsy pathological. "-", No follow-up result.

## Literature reviews

We searched PubMed and MEDLINE for studies on DM with PCI using the keywords “pneumatosis cystoides intestinalis” or “pneumatosis intestinalis” and “dermatomyositis” in English from 1960 to 2022. Literature that clearly diagnosed DM and excluded juvenile DM with PCI was reviewed.

A total of 11 unique case reports and series, including 12 adult DM patients with PCI, were identified. Of the 12 patients, 11 were women (91.7%), and 1 was a man (8.3%). The age at disease onset ranged from 30 to 70 years, with a median age of 58 years. Only one patient had detected myositis-specific antibodies (MSA) in all cases. When these patients were diagnosed with PCI, six patients were in a state of disease activity, and the remaining patients were in stable condition. All patients were treated with glucocorticoids (GCs) and disease-modifying antirheumatic drugs (DMARDs). Ten patients had the symptoms at the time of PCI onset, including abdominal pain, abdominal distension, fever, diarrhea, nausea, and vomiting. The colon was the most common site of PCI involvement. Of the 12 patients, two received surgical treatment, while the others received treatment, including fasting, antibiotics, oxygen therapy, and observation. Histological examination was performed in six patients, and no vasculitis was observed. Two patients died due to infection and acute respiratory distress syndrome (ARDS), respectively. The clinical data of the 12 patients are shown in **Table 1** (7, 11–20).

## Discussion

PCI is a rare disease characterized by the presence of inflatable cysts in the intestinal wall. It may involve the entire GI tract, especially the small intestine and colon. PCI was first recorded by Duvernoi at an autopsy in 1730 (21). PCI can occur in many autoimmune diseases, such as systemic sclerosis (SSc) and systemic lupus erythematosus (SLE) (22). However, few articles have reported PCI in DM. In this report, we describe in detail the manifestation and prognosis of PCI in DM.

The pathological mechanism of PCI is unclear. Several hypotheses have been proposed in autoimmune diseases. The first reason is digestive dysfunction. In many autoimmune diseases, such as SSc, smooth muscle cells in the GI wall undergo inflammation, atrophy, degeneration, and fibrosis. These changes can lead to decreased digestive function, leading to gastroparesis or constipation. Excessive bacterial growth and intestinal expansion can cause increased pressure within the lumen, which leads to gas entering the intestinal wall in this condition (23, 24). The second reason is intestinal vasculitis. Intestinal vasculitis can cause damage to the mucosa membrane and immune barrier. Mizoguchi et al. summarized the results of 14 SLE patients with PCI. The pathological analysis showed that six out of seven patients had vasculitis. Therefore, unlike SSc, the pathogenesis of SLE complicated by PCI may be intestine vasculitis (25). The third one is drugs. It has been suggested that GC, DMARDs, alpha-glucosidase inhibitors (AGIs), cytotoxic anticancer drugs, and molecular-targeted drugs are associated with PCI (26–31). GC and DMARDs are commonly used in autoimmune diseases. It has been reported that GC may deplete the lymphoid tissue within the Payer’s patches, resulting in mucosal disruption and intraluminal gas diffusion into the intestinal wall. Additionally, GC administration can sometimes induce diabetes mellitus, and AGIs are also used to control glucose levels. Increased intraluminal pressure caused by AGIs can also lead to gas dissection into the intestinal wall. The last reason is bacterial fermentation within the lumen. Bacteria can enter the intestinal wall through increased mucosal permeability, decompose nutrients in the intestinal wall, and generate gas, resulting in intestinal pneumatosis.

The etiology of PCI in patients with DM is still unknown. In this case series, all patients received GC and DMARD treatment. Out of the six patients, five took acarbose to control diabetes, which has been reported to be related to PCI. However, they did not discontinue these drugs, and four of them recovered during the follow-up period. The patients in our cohort developed PCI during both the active and stable stages of the disease. Therefore, whether disease activity affects the occurrence of PCI is unclear. Considering that the cause of PCI in patients with SLE may be related to vasculitis, we also believe that some patients with DM may have PCI associated with vasculitis. Furthermore, mediastinal emphysema is relatively common in DM, particularly in patients with anti-MDA5 antibodies (32). This condition can cause gas to enter the intestinal wall along the aorta and mesenteric vessels (33). In our cases, two patients experienced mediastinal emphysema. These findings prove that PCI development in DM is complex.

However, some issues need special attention in DM. Out of the six patients, four had anti-MDA5 antibodies. This may be due to selection bias. Patients with DM in our center were mainly with anti-MDA5 antibodies. However, complete and partial MDA5 deficiency has been reported to be associated with inflammatory bowel disease (IBD) (34). Previous literature has reported that PCI was associated with IBD (35). Therefore, it is worth exploring whether MDA5 is related to the emergence of PCI. In our cohort, PCI in all patients was located in the ascending colon. This is similar to a previous report that showed that approximately 80% of asymptomatic PCI occurred in the ascending colon (36). Fortunately, only one patient experienced temporary abdominal symptoms, whereas the remaining patients were completely asymptomatic. No patients had serious complications without PCI-related treatment. Although PCI is a benign disease in most cases, severe complications of PCI such as perforation and death have been reported. Notably, this harmless PCI as “isolated PCI” might be different from severe bowel vasculitis in DM.

In conclusion, PCI is an uncommon complication in adult patients with DM. The development of PCI in adult patients with DM may be related to multiple factors. The symptoms of PCI in DM are usually non-specific, and most patients have no serious complications without further treatment.

## Data availability statement

The original contributions presented in the study are included in the article/supplementary material. Further inquiries can be directed to the corresponding author.

## Ethics statement

The studies involving human participants were reviewed and approved by the Ethics Committee of China-Japan Friendship Hospital. The patients/participants provided their written informed consent to participate in this study. Written informed consent was obtained from the participant/patient(s) for the publication of this case report.

## Author contributions

JL and SL designed the study and wrote the manuscript. LZ provided all the images in the article. SC participated in the assessment of the digestive system. XL supervised and revised the manuscript. All authors contributed to the article and approved the submitted version.
